# Risk factors for stunting in children who are HIV‐exposed and uninfected after Option B+ implementation in Malawi

**DOI:** 10.1111/mcn.13451

**Published:** 2022-11-09

**Authors:** Gabriela Toledo, Megan Landes, Monique van Lettow, Beth A. Tippett Barr, Heather Bailey, Siobhan Crichton, Wezi Msungama, Claire Thorne

**Affiliations:** ^1^ Population, Policy, and Practice Research & Teaching Department, Great Ormond Street Institute of Child Health University College London London UK; ^2^ Department of Family and Community Medicine University of Toronto Toronto Ontario Canada; ^3^ Dalla Lana School of Public Health University of Toronto Toronto Ontario Canada; ^4^ Nyanja Health Research Institute Salima Malawi; ^5^ Institute for Global Health, University College London London UK; ^6^ Medical Research Council Clinical Trials Unit University College London London UK; ^7^ Division of Global HIV and Tuberculosis Centers for Disease Control and Prevention Lilongwe Malawi

**Keywords:** ART, child, growth, HIV, Malawi, Option B+, stunting

## Abstract

Evidence suggests children HIV‐exposed and uninfected (CHEU) experience poor growth. We analysed child anthropometrics and explored factors associated with stunting among Malawian CHEU. Mothers with HIV and their infants HIV‐exposed were enroled in a nationally representative prospective cohort within the National Evaluation of Malawi's Prevention of Mother‐to‐Child HIV Transmission Programme after Option B+ implementation (2014–2018). Anthropometry was measured at enrolment (age 1–6 months), visit 1 (approximately 12 months), and visit 2 (approximately 24 months). Weight‐for‐age (WAZ) and length‐for‐age (LAZ) z‐scores were calculated using World Health Organization Growth Standards; underweight and stunting were defined as WAZ and LAZ more than 2 standard deviations below the reference median. Multivariable logistic regression restricted to CHEU aged 24 months (±3 months) was used to identify factors associated with stunting. Among 1211 CHEU, 562/1211 attended visit 2, of which 529 were aged 24 months (±3 months) and were included. At age 24 months, 40.4% of CHEU were stunted and/or underweight, respectively. In multi‐variable analysis, adjusting for child age and sex, the odds of stunting were higher among CHEU with infectious disease diagnosis compared to those with no diagnosis (adjusted odds ratio = 3.35 [95% confidence interval: 1.82–6.17]), which was modified by co‐trimoxazole prophylaxis (*p* = 0.028). Infant low birthweight was associated with an increased odds of stunting; optimal feeding and maternal employment were correlated with reduced odds. This is one of the first studies examining CHEU growth since Option B+. Interventions to improve linear growth among CHEU should address their multi‐faceted health risks, alongside maternal ART prescription, and follow‐up of mother‐child pairs.

## Key messages

- Understanding the health risks of children HIV‐exposed and uninfected (CHEU) and the impact on their growth is of paramount importance for optimizing their health.
- This is one of the first studies examining correlates of CHEU growth since the implementation of Option B+ and uses a random subsample of a nationally representative cohort of women with HIV and CHEU in Malawi.
- Factors associated with stunting included infant low birthweight, optimal feeding, co‐trimoxazole prophylaxis, and maternal employment.
- Interventions targeting modifiable risk factors, alongside maternal antiretroviral therapy, and improved nutritional follow‐up of CHEU will contribute to improving CHEU growth in Malawi.

## Graphical abstract

Mothers with HIV and their infants HIV‐exposed were enroled in a prospective cohort within the National Evaluation of Malawi's Prevention of Mother‐to‐Child HIV Transmission Programme after Option B+ implementation (2014–2018). Weight‐for‐age (WAZ) and length‐for‐age (LAZ) z‐scores were calculated using World Health Organization Growth Standards; underweight and stunting were defined as WAZ and LAZ more than 2 standard deviations below the reference median. Multi‐variable logistic regression restricted to CHEU aged 24 months (±3 months) was used to identify factors associated with stunting. In multi‐variable analysis, adjusting for child age and sex, the odds of stunting were higher among CHEU with infectious disease diagnosis compared to those with no diagnosis (adjusted odds ratio = 3.35 [95% confidence interval: 1.82–6.17]), which was modified by receipt of co‐trimoxazole prophylaxis (*p* = 0.028). Infant low birthweight was associated with an increased odds of stunting; optimal feeding and maternal employment were correlated with reduced odds.

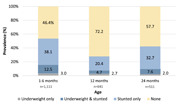

## 1 INTRODUCTION

In 2020, an estimated 15.4 million children under the age of 14 were HIV‐exposed and uninfected (CHEU) globally, with 13.8 million living in sub‐Saharan Africa (UNAIDS, 2021). Understanding the health risks of CHEU and the impact on their growth is essential for improving child health in sub‐Saharan Africa.

Stunting is an abnormally slow rate of gain in a child's length or height, which typically begins at birth and continues through 2 years of age, and has far‐reaching consequences on the whole cycle of child growth and development (Adair et al., 2013; Black et al., 2008; Cheung & Ashorn, 2010; Olofin et al., 2013). In South Africa, two large cohort studies demonstrated breastfed ART‐exposed CHEU present with lower length‐for‐age (LAZ) z‐scores and greater prevalence of stunting than children HIV‐unexposed (Evans et al., 2021; S. M. Le Roux et al., 2019), with similar findings for weight‐for‐age (WAZ) z‐scores and underweight (Evans et al., 2021). In a longitudinal analysis of ART‐exposed Malawian CHEU, median LAZ and WAZ were lower through 60 months of age compared to children HIV‐unexposed (Fowler et al., 2021).

With widespread availability of antiretroviral therapy (ART) for prevention of vertical transmission (PVT) and treat‐all policies (i.e., universal treatment of HIV), ART use among pregnant WLHIV has increased substantially. While this has improved maternal health and survival, partially mitigating disparities in morbidity and mortality between CHEU and children HIV‐unexposed (Brennan et al., 2016; S. M. Le Roux et al., 2016), available evidence suggests poor growth persists as a problem for CHEU (Aizire et al., 2020; Evans et al., 2021; Fowler et al., 2021; Jumare et al., 2019; S. M. Le Roux et al., 2019; Ndiaye et al., 2021; Sudfeld et al., 2016). Although ART use during pregnancy is generally safe, specific antiretrovirals have been associated with intrauterine growth restriction and preterm birth (Uthman et al., 2017). As such, research is needed to identify which HIV‐specific exposures or universal risk factors are growth discriminatory among CHEU in the universal ART era.

Restricted linear growth in early childhood is often caused by the interplay of child undernutrition, increased exposure to infectious agents, and maternal morbidity (Black et al., 2013; Engle et al., 2011; Olofin et al., 2013; Slogrove et al., 2020; Walker et al., 2011; Wedderburn et al., 2019). Suboptimal nutrition and exposure to HIV may have downstream effects on early child growth by disrupting normal microbial succession (i.e., assembly of microbial communities within the gastrointestinal tract; D. M. Le Roux et al., 2019; Shafiq et al., 2021; Yeates et al., 2020). As infants born to women living with HIV (WLHIV) are recommended co‐trimoxazole prophylaxis (CTX) from age 6 weeks until HIV infection has been ruled out, to prevent opportunistic infections that are known risk factors for poor child growth (World Health Organization, 2021), this may reduce CHEU risk of stunting. To date, findings on the protective effects of CTX against common childhood illnesses including malaria and diarrhoea are mixed.

High levels of malnutrition are endemic in Malawi, where an estimated 37% of children under 5 are stunted and 12% are underweight (The DHS Programme, 2017). It has one of the highest HIV prevalence rates globally, with 9% of adults aged 15–49 years living with HIV (UNAIDS, 2021). As the first country to recommend Option B+, Malawi is recognised as a pioneer in PVT (World Health Organization, 2013), where HIV Care Clinics (HCCs) offer integrated services for families affected by HIV and tailored care for CHEU. Annually, ART coverage for pregnant WLHIV is estimated at 98% for those engaged in care (Malawi, 2018; UNAIDS, 2020), approximately 90% of WLHIV at 1–6 months postpartum are ART adherent (Van Lettow et al., 2021), and about 7% of the total paediatric population (aged 0–14 years) is HIV‐exposed and uninfected (UNAIDS, 2020). Together, this makes Malawi a unique and important setting to study CHEU growth outcomes.

Currently, there are limited and inconsistent data on the growth of CHEU and the factors potentially driving their stunted growth in the context of maternal lifelong ART. Here we describe the growth patterns of CHEU in Malawi and identify maternal, child, and contextual factors associated with stunting among CHEU aged 24 months.

## 2 METHODS

### 2.1 Study design and participants

This study is based on a nationally representative prospective cohort within the “National Evaluation of Malawi's Prevention of Mother‐to‐Child Transmission of HIV Programme (NEMAPP)” Study which was designed to obtain data on maternal ART coverage and PVT effectiveness after Option B+ implementation. NEMAPP enroled infants HIV‐exposed aged 1–6 months and their mothers at under‐5 outpatient clinics in Malawi (2018), using a multi‐stage cluster sampling design to randomly sample 54 health facilities across rural and urban Malawi (Tippett Barr et al., 2018; Van Lettow et al., 2018). At each health facility, mothers provided written informed consent for screening; mothers with a positive rapid HIV test and a positive HIV polymerase chain reaction (PCR) test result were invited to participate in the study and provided consent for infant enrolment in the cohort. At the time of the study, pregnant and breastfeeding WLHIV were started on lifelong ART (daily fixed‐dose tenofovir [TDF] + lamivudine [3TC] + efavirenz [EFV]).

Following study enrolment at age 1–6 months, follow‐up visit 1 was at approximately age 12 months and visit 2 was at approximately age 24 months (scheduled to coincide with mothers' routine ART appointments). At study visits, mothers/guardians were interviewed using structured questionnaires by on‐site trained study staff to collect information on maternal, child, and contextual factors. For a nested subset of mother–child pairs from 13 of the 54 health facilities sampled (long‐term follow‐up cohort), weight, length, and mid‐upper arm circumference were measured and blood samples were collected at enrolment, visit 1, and visit 2. Virological markers for the long‐term follow‐up cohort (e.g., maternal HIV viral load) were obtained from laboratory testing on blood samples collected during study visits.

### 2.2 Inclusion criteria for analysis

Inclusion criteria for this analysis included singleton births only and children confirmed HIV‐uninfected by a negative PCR test result at each study visit.

### 2.3 Outcomes

Outcomes of interest were WAZ and LAZ generated using the WHO Growth Standards with child age recorded in whole months (World Health Organization, 2006). Estimates for WAZ and LAZ at 12 months were restricted to CHEU aged 12 months (±3 months) at visit 1 and estimates at 24 months were restricted to CHEU aged 24 months (±3 months) at visit 2. Underweight and stunting were respectively defined as WAZ and LAZ more than 2 standard deviations (SD) below the reference median (World Health Organization, 2006).

### 2.4 Investigated exposures

Exposures were selected based on the literature and hypothesised relationships. Maternal characteristics (e.g., age, timing of ART initiation and maternal HIV viral load), child characteristics (e.g., birthweight, diagnosis of an infectious disease, receipt of CTX), and contextual factors (e.g., place of birth, education and employment) were considered. Exposures were mother‐reported unless noted otherwise; markers for maternal health, birth outcomes, HIV care uptake, and mother–child pair outcomes were validated using mothers' health booklets and confirmed using Ministry of Health registers when possible.

Child feeding was assessed at all visits with 1‐week recall; mothers were asked about liquid and food intake (e.g. breast milk, non‐breast milk, sobo, soup, fruit, porridge, plumpy nut, vegetables, eggs, meat, fish, nsima and other). Exclusive breastfeeding was defined as exclusive breast milk consumption in the past 7 days at enrolment. Optimal feeding at age 24 months was defined as any mother‐reported milk feeding (e.g., breast or non‐breast milk) plus semi‐solid and/or solid foods in the past 7 days from at least three food groups (carbohydrates; fruits/vegetables; legumes and nuts; eggs; and flesh foods), based on definitions in the literature (FANTA, 2007; World Health Organization, 2008).

Markers for child morbidity included diagnosis of an infectious disease, defined as any diagnosed episode of malaria, diarrhoea, pneumonia, meningitis, and/or tuberculosis in the previous 3 months at enrolment (or birth for children aged <3 months at enrolment) or since the previous study visit; child ever hospitalised; and number of sick visits to a health facility or clinic, defined as 0 times, 1‐2 times, or ≥3 times in the previous 3 months (or birth for children aged <3 months at enrolment).

Where indicated, continuous and discrete data were categorised according to the data and standard or published boundaries, including infant birthweight (low birthweight defined as <2.5 kg), maternal age (<21, 21–34 and ≥35 years), maternal mid‐upper arm circumference (MUAC; underweight defined as a MUAC < 24 cm), and maternal antenatal care visits (<4 or ≥4 visits).

### 2.5 Statistical analysis

Descriptive statistics were used to summarise enrolment characteristics and child anthropometry at age 1–6, 12, and 24 months. Enrolment characteristics were compared between CHEU who did and did not complete follow‐up visits to age 24 months to examine differences between those that were and were not included in the multi‐variable analysis. We hypothesised that CTX may have a protective effect against stunting (Sandison et al., 2011; World Health Organization, 2021) through effect modification on the association between child morbidity and stunting; markers for child morbidity were compared by receipt of CTX at age 24 months.

Multi‐variable logistic regression allowing for clustering by health facility was used to identify maternal, child, and/or contextual factors associated with stunting at age 24 months (±3 months), adjusted for child age and sex. As linear growth faltering is cumulative from birth to 2 years of age, multi‐variable models were restricted to CHEU aged 24 months at last follow‐up. Covariates with *p* < 0.3 in univariate analysis were considered for inclusion in the multi‐variable model. Where multiple markers were available for the same construct, variables were selected based on the literature and model fit indices. Backward elimination was applied and variables with *p* < 0.1 were retained in the model. In a stepwise fashion, covariates dropped after backward elimination were added to the final model to check for statistical significance at *p* < 0.1. Potential effect modification by receipt of CTX on the association between child morbidity and stunting was evaluated; hypothesised interactions were fitted in the final model using forward selection if *p* < 0.1. All analyses were conducted on Stata MP version 17 (Stata Corp., College Station, TX).

### 2.6 Sensitivity analysis

Two restricted analyses were conducted to assess the robustness of our findings. First, we described child anthropometry in CHEU with WAZ and/or LAZ available at all study visits (i.e., complete case). Second, as child age was only available in whole months, growth z‐scores were recalculated using child age minus 2 weeks to estimate minimum prevalence rates for underweight and stunting by child age.

As a large proportion of mother–child pairs did not attend follow‐up at age 24 months, we computed inverse probability weights (IPWs) to adjust for potential selection bias. Each child who attended follow‐up at 24 months received a weight inversely proportional to the estimated probability of attending follow‐up at 24 months. Weights were computed using logistic regression and enrolment covariates; covariates were selected *a priori* or were predictors of follow‐up and/or stunting at 24 months. The model fitting procedure described above was then repeated using the weighted data. As a backward elimination procedure was used, variables with borderline significant *p* values may have been included in one model and excluded in the other. Where this occurred, odds ratios and *p*‐values for excluded variables were calculated by adding each variable one at a time to the final models.

### 2.7 Ethics statement

Ethical approval for the NEMAPP Study was obtained from Malawi National Health Sciences Research Committee (NHSRC, #1262), the US Centers for Disease Control and Prevention Global Health Associate Director for Science (#2014‐054‐7), and the University of Toronto Research Ethics Committee (#30448). Ethical approval for this study was obtained from University College London (UCL) Research Ethics Committee (Ref: 3715/004).

## 3 RESULTS

### 3.1 Enrolment characteristics

**Figure 1:**
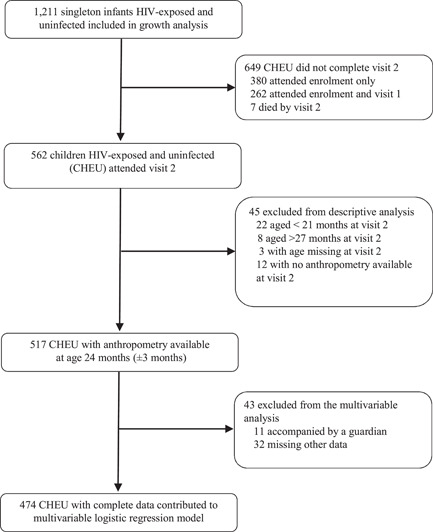
Study flow diagram

Overall, 1211 singleton CHEU from 13 health facilities (long‐term follow‐up cohort) were enroled; 1206 were accompanied by their mothers and the remaining (*n* = 5) presented with a guardian. Of 1211 mother–child pairs enroled, 566 (46.7%) mothers (or guardians) and 562 (46.7%) CHEU completed follow‐up through 24 months of age (Figure 1). Maternal, contextual, and child characteristics at enrolment are presented in Tables 1a, 1b. The maternal median age at enrolment was 29 years (interquartile range [IQR]: 24–33); approximately half of the mothers initiated ART before pregnancy and almost all mothers were on ART at the time of enrolment (Table 1a). Maternal viral load for almost a quarter of mothers was detectable (≥40 copies/ml) at enrolment and one fifth of mothers were underweight based on maternal MUAC (Table 1a). For CHEU, median age at enrolment was 2 months (IQR: 2–4), 91.4% were exclusively breastfed in the past 7 days, and about 60.0% were enroled in HCCs (Table 1b). Median birthweight was 3.0 kg (IQR: 2.7–3.4) and 11.8% had low birthweight. CHEU received nevirapine prophylaxis for a mean of 40.3 days (SD: 6.7) out of the 42 days recommended as prophylaxis for infants born to WLHIV, and among those aged ≥2 months at enrolment (*n* = 1199), nearly half were receiving CTX (Table 1b).

**Table 1a:** Enrolment maternal characteristics and contextual factors

	Cohort	MCPs without 24 months FUP	MCPs with 24 months FUP	*p* Value	Total
	*N* = 1211	*N* = 645	*N* = 566		1211
Median [IQR], mean {SD} or *n* (%)					
**Maternal characteristics** a					
Age (years)	29.0 [24.0–33.0]	28.0 [23.0–32.0]	30.0 [25.0–34.0]	<0.001	1207
Parity	3.1 {1.5}	2.9 {1.5}	3.3 {1.5}	<0.001	1211
Number of antenatal care visits	3.5 {1.1}	3.4 {1.1}	3.5 {1.0}	0.027	1199
Ever received ARVs for PVT in previous pregnancy^b^				0.820	1184
No	681 (57.5%)	361 (57.2%)	320 (57.9%)		
Yes	503 (42.5%)	270 (42.8%)	233 (42.1%)		
Maternal‐reported health at study enrolment				0.140	1201
No illness	1143 (95.2%)	615 (96.1%)	528 (94.1%)		
A little bit sick	50 (4.2%)	20 (3.1%)	30 (5.3%)		
Very sick	8 (0.7%)	5 (0.8%)	3 (0.5%)		
HIV status at study enrolment				<0.001	1205
Already known positive	1155 (95.9%)	603 (93.9%)	552 (98.0%)		
Newly diagnosed positive through study	50 (4.1%)	39 (6.1%)	11 (2.0%)		
Timing of ART initiation				<0.001	1185
Pre‐conception	570 (48.1%)	283 (44.8%)	287 (51.9%)		
1st/2nd trimester	475 (40.1%)	255 (40.3%)	220 (39.8%)		
3rd trimester/postpartum	140 (11.8%)	94 (14.9%)	46 (8.3%)		
Current ART use at study enrolment				0.140	1197
No, never on ART	3 (0.3%)	2 (0.3%)	1 (0.2%)		
No, stopped ART	11 (0.9%)	9 (1.4%)	2 (0.4%)		
Yes, on ART now	1,183 (98.8%)	626 (98.3%)	557 (99.5%)		
HIV viral load at study enrolment				<0.001	1176
VL detectable	272 (23.1%)	183 (29.2%)	89 (16.2%)		
VL undetectable	904 (76.9%)	443 (70.8%)	461 (83.8%)		
Suboptimal ART adherence in past 30 days				0.006	1127
Adherent	987 (87.6%)	497 (85.0%)	490 (90.4%)		
Non‐adherent (≥2 days missed)	140 (12.4%)	88 (15.0%)	52 (9.6%)		
Mid‐upper arm circumference				0.270	882
Normal	706 (80.0%)	370 (81.5%)	336 (78.5%)		
Underweight (<24 cm)	176 (20.0%)	84 (18.5%)	92 (21.5%)		
**Contextual characteristics** a					
Maternal spouse or partner				0.950	1202
No	68 (5.7%)	36 (5.6%)	32 (5.7%)		
Yes	1134 (94.3%)	605 (94.4%)	529 (94.3%)		
Maternal education				0.94	1208
None	104 (8.6%)	53 (8.3%)	51 (9.0%)		
Primary education	684 (56.6%)	368 (57.3%)	316 (55.8%)		
Secondary education	398 (32.9%)	209 (32.6%)	189 (33.4%)		
Postsecondary education	22 (1.8%)	12 (1.9%)	10 (1.8%)		
Maternal employment^c^				0.970	1201
Not formally employed	790 (65.8%)	422 (65.8%)	368 (65.7%)		
Employed	411 (34.2%)	219 (34.2%)	192 (34.3%)		
Geographical region				<0.001	1206
Blantyre Urban	376 (31.2%)	200 (31.1%)	176 (31.3%)		
Lilongwe Urban	364 (30.2%)	237 (36.9%)	127 (22.6%)		
North and Central rural	317 (26.3%)	164 (25.5%)	153 (27.2%)		
South rural	149 (12.4%)	42 (6.5%)	107 (19/0%)		
Travel time from home to clinic				0.920	1193
<1 h	643 (53.9%)	346 (54.2%)	297 (53.5%)		
1–2 h	451 (37.8%)	238 (37.3%)	213 (38.4%)		
≥2 h	99 (8.3%)	54 (8.5%)	45 (8.1%)		

Abbreviations: ART, antiretroviral therapy; ARV, antiretroviral; FUP, follow‐up; MCP, mother–child pair; PVT, prevention of vertical transmission; VL, viral load.

^a^ 5/1211 CHEU were accompanied by a guardian at study enrolment; for these MCPs, maternal data were only available on age, parity, antenatal care visits, and education.

^b^ Ever received antiretrovirals for a pregnancy before the most recent pregnancy with study child.

^c^ Not formally employed included mothers who self‐reported their employment as ‘housewife’.

**Table 1b:** Enrolment child characteristics

	Cohort	MCPs without 24 months FUP	MCPs with 24 months FUP		Total
	*N* = 1211	*N* = 645	*N* = 566^a^	*p* Value	1211
Median [IQR], mean {SD}, or *n* (%)					
**Child characteristics**					
Age (months)	2.0 [2.0–4.0]	2.0 [2.0–4.0]	2.0 [2.0–4.0]	0.680	1211
Sex				0.320	1211
Male	611 (50.5%)	334 (51.8%)	277 (48.9%)		
Female	600 (49.5%)	311 (48.2%)	289 (51.1%)		
Born in health facility or clinic				0.630	1206
No	55 (4.6%)	31 (4.8%)	24 (4.3%)		
Yes	1151 (95.4%)	611 (95.2%)	540 (95.7%)		
Low birthweight				0.536	1163
No	1027 (88.3%)	550 (88.8%)	477 (87.7%)		
Yes	136 (11.7%)	69 (11.2%)	67 (12.3%)		
Exclusive breastfeeding in past 7 days				0.048	1211
No	104 (8.6%)	65 (10.1%)	39 (6.9%)		
Yes	1107 (91.4%)	580 (89.9%)	527 (93.1%)		
Number of sick visits to health facility or clinic				0.570	1203
0 times	958 (79.6%)	504 (78.5%)	454 (80.9%)		
1 time	189 (15.7%)	107 (16.7%)	82 (14.6%)		
≥2 times	56 (4.7%)	31 (4.8%)	25 (4.5%)		
Diagnosis of an infectious disease^b^				0.860	1205
No	1133 (94.0%)	602 (93.9%)	531 (94.1%)		
Yes	72 (6.0%)	39 (6.1%)	33 (5.9%)		
Ever been admitted to hospital				0.919	1207
No	1,148 (95.1%)	611 (95.2%)	537 (95.0%)		
Yes	59 (4.9%)	31 (4.8%)	28 (5.0%)		
Immunised for Hepatitis B^c^				0.415	1169
No	175 (15.0%)	88 (14.2%)	87 (15.9%)		
Yes	994 (85.0%)	533 (85.8%)	461 (84.1%)		
Days child received nevirapine syrup	40.3 {6.7}	40.1 {7.2}	40.6 {6.2}	0.240	1133
Enroled in HIV Care Clinic				0.015	1200
No	511 (42.6%)	292 (45.8%)	219 (38.9%)		
Yes	689 (57.4%)	345 (54.2%)	344 (61.1%)		
Received co‐trimoxazole prophylaxis^c^				0.047	1147
No	588 (51.3%)	329 (54.0%)	259 (48.1%)		
Yes	559 (48.7%)	280 (46.0%)	279 (51.9%)		
Anthropometry					
Weight‐for‐age z‐score	−0.70 [−1.5, 0.2]	−0.6 [−1.5, 0.2]	−0.70 [−1.5, 0.1]	0.390	1189
Length‐for‐age z‐score	−2.1 [−3.3, −1.0]	−2.1 [−3.5, −1.0]	−2.1 [−3.2, −1.0]	0.250	1119

Abbreviations: FUP, follow‐up; MCP, mother–child pair.

^a^ 566 mothers (or guardians) and 562 CHEU attended follow‐up at 24 months; 4/566 (0.71%) mothers reported child deaths at 24 months.

^b^ Among 1205 CHEU with data available on diagnosis of an infectious disease, 1.0%, 1.8%, 3.5%, 0.1% and 0.2% were reported to have malaria, diarrhoea, pneumonia, meningitis and/or tuberculosis in the previous 3 months (or since birth for infants aged less than 3 months at study enrolment), respectively.

^c^ Restricted to children aged ≥2 months at study enrolment.

There were some differences in enrolment characteristics between CHEU completing study visits to age 24 months and those who did not (Tables 1a, 1b). Most notably, CHEU not completing follow‐up had younger mothers, with fewer antenatal care visits, who were more likely to be newly diagnosed with HIV through the NEMAPP study, and more likely to have a detectable viral load. CHEU not completing follow‐up were less likely to be enroled in HCCs and receiving CTX; exclusive breastfeeding was similar in both groups. At age 24 months, of 1211 CHEU enroled in the long‐term follow‐up cohort, 562 (46.4%) children remained alive and HIV‐free, 7 (0.6%) died, and 642 (48.9%) did not attend follow‐up at 24 months (transferred out or loss‐to‐follow‐up). Among CHEU (*n* = 11) who were accompanied by a guardian at 24‐month visit, 54.5% (6/11) reported maternal deaths.

### 3.2 Anthropometry by child age

**Figure 2:**
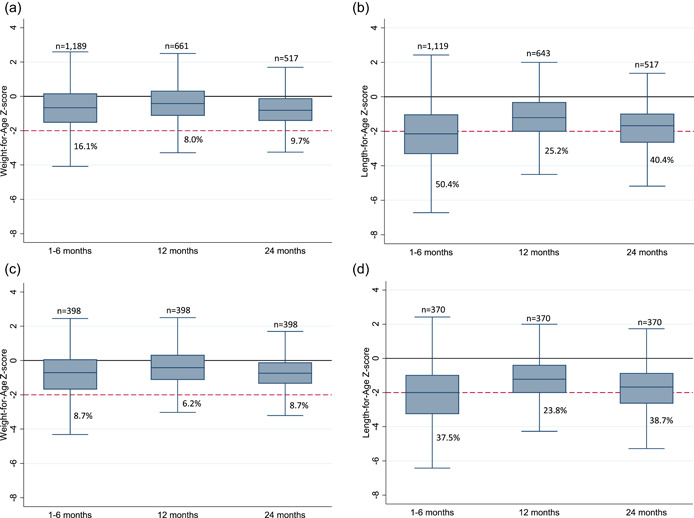
(a, b) Weight‐for‐age (WAZ) and length‐for‐age (LAZ) median z‐scores for whole cohort of CHEU by child age; (c, d) WAZ and LAZ median z‐scores for complete‐cases by child age. *Y*‐line at 0 represents median WAZ and LAZ for the reference population, and *y*‐line at −2 represents underweight (a–c) or stunted growth (b–d); percentages constitute estimated prevalence rates for underweight (a–c) or stunted growth (b–d) by child age.

Among 1211 CHEU at enrolment, 98.2% had WAZ and/or LAZ available; 743/1211 (61.4%) attended visit 1, of which 667 were age 9–15 months at the visit and were included (96.4% had anthropometry available), and 562/1211 (46.7%) attended visit 2, of which 529 were age 21–27 months at the visit and were included (97.7% had anthropometry available). From enrolment to age 12 months, there was some evidence of early catch‐up in WAZ and LAZ, although this was not prolonged, as z‐scores declined by 24 months (Supporting Information: Table S1, Figure 2). Among 398 and 370 CHEU with WAZ and/or LAZ respectively available at all study visits, patterns in growth were consistent with patterns observed in the whole cohort (Supporting Information: Table S1, Figure 2).

**Figure 3:**
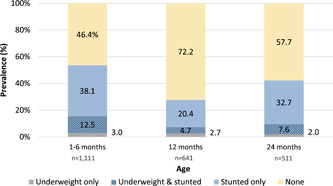
Overall prevalence of growth deficiencies among CHEU with both WAZ and LAZ available by child age.

At age 24 months, 9.7% of CHEU were underweight and 40.4% were stunted (Figure 2). In our sensitivity analysis, the difference in prevalence estimates for stunting at 24 months was negligible (less than 2%; Supporting Information: Table S2). Among CHEU with both WAZ and LAZ available at 24 months, 2.0% of CHEU were underweight only, 7.6% were underweight and stunted, 32.7% were stunted only, and 57.7% had no growth deficiencies (Figure 3).

### 3.3 Factors associated with stunting at 24 months of age

Overall, 474 CHEU with complete data across the exposure variables of interest contributed to the risk factor analysis in Table 2 and the multi‐variable analysis on stunting in Table 3. At age 24 months, there was no association between receipt of CTX and CHEU diagnosis of an infectious disease (39.7% vs. 32.3%, *p* = 0.183) or ever being hospitalised (15.3% vs. 20.8%, *p* = 0.195).

**Table 2:** Risk analysis of stunted growth

			Unadjusted OR (95% CI)	
	Total (*N*)	*n* (%) stunted	*N* = 474	*p* Value
**Child characteristics**				
Age (months)	474		1.21 (1.06–1.38)	0.004
Sex				
Male	232	104 (44.8%)	Ref	
Female	242	87 (36.0%)	0.69 (0.49–0.98)	0.038
Born in health facility or clinic				
No	15	5 (33.3%)	Ref	
Yes	457	186 (40.7%)	1.37 (0.45–4.17)	0.577
Low birthweight				
No	415	163 (38.8%)	Ref	
Yes	59	30 (50.9%)	1.63 (1.10–2.43)	0.016
Exclusive breastfeeding in the past 7 days at enrolment				
No	32	18 (56.3%)	Ref	
Yes	442	173 (39.1%)	0.50 (0.24–1.04)	0.063
Any milk feeding in the past 7 days				
None	214	96 (44.9%)	Ref	0.133
Breast milk	29	9 (31.0%)	0.55 (0.27–1.12)	
Non‐breast milk	231	86 (37.2%)	0.73 (0.51–1.03)	
Optimal feeding in the past 7 days				
No	215	96 (44.7%)	Ref	
Yes	259	95 (36.7%)	0.72 (0.50–1.02)	0.068
Parent‐reported child health at 24 months				
Sick	16	6 (37.5%)	Ref	
Well	458	185 (40.4%)	1.13 (0.47–2.72)	0.786
Diagnosis of an infectious disease^a^				
No	293	107 (36.5%)	Ref	
Yes	181	84 (46.4%)	1.51 (1.20–1.89)	<0.001
Number of sick visits to a health facility or clinic				
0 times	301	113 (37.5%)	Ref	0.001
1 time	121	52 (43.0%)	1.25 (0.84–1.87)	
≥2 times	52	26 (50.0%)	1.66 (1.01–2.73)	
Ever been hospitalised				
No	396	152 (38.4%)	Ref	
Yes	78	39 (50.0%)	1.61 (0.98–2.62)	0.058
Received co‐trimoxazole prophylaxis at 24 months				
No	96	50 (52.1%)	Ref	
Yes	378	141 (37.3%)	0.55 (0.37–0.80)	0.002
**Maternal characteristics**				
Age				
<21 years	34	20 (58.8%)	Ref	0.087
21‐34 years	332	128 (38.6%)	0.44 (0.20–0.95)	
≥35 years	108	43 (39.8%)	0.46 (0.18–1.20)	
Parity				
Primiparous	58	23 (39.7%)	Ref	
Multiparous	416	168 (40.4%)	1.03 (0.60–1.78)	0.913
Number of antenatal care visits during pregnancy				
<4 visits	229	88 (38.4%)	Ref	
≥4 visits	240	100 (41.7%)	1.14 (0.82–1.59)	0.420
Timing of ART initiation				
Pre‐conception	239	99 (41.4%)	1.10 (0.65–1.88)	
1st/2nd trimester	187	73 (39.0%)	1.00 (0.65–1.54)	
3rd trimester/postpartum	41	16 (39.0%)	Ref	0.842
Maternal‐reported health at ART initiation				
No illness	401	161 (40.2%)	Ref	0.575
A little bit sick	56	24 (42.9%)	1.12 (0.90–1.40)	
Very sick	17	6 (35.3%)	0.81 (0.17–3.90)	
Current ART use at 24 months				
No, never on ART	–	–	–	
No, stopped ART	–	–	–	
Yes, on ART now	474	191 (40.3%)	–	
HIV viral load at study enrolment				
Detectable	73	32 (43.8%)	Ref	
Undetectable	390	156 (40.0%)	0.85 (0.51–1.42)	0.543
HIV viral load at 24 months				
Detectable	50	22 (44.0%)	Ref	
Undetectable	386	156 (40.4%)	0.86 (0.45–1.65)	0.658
Ever used ARVs for PVT in previous pregnancy^b^				
No	264	105 (39.8%)	Ref	
Yes	203	84 (41.4%)	1.07 (0.82–1.39)	0.615
Suboptimal ART adherence in the past 30 days				
Adherent	444	173 (39.0%)	Ref	0.039
Non‐adherent (≥ 2 days missed)	30	18 (60.0%)	2.35 (1.04–5.30)	
Maternal‐reported health at 24 months				
No illness	463	188 (40.6%)	Ref	
A little bit sick	11	3 (27.3%)	0.55 (0.12–2.42)	0.427
Mid‐upper arm circumference at 24 months				
Normal	409	159 (38.9%)	Ref	
Underweight (<24 cm)	65	32 (49.2%)	1.52 (0.84–2.76)	0.162
**Contextual factors**				
Maternal spouse				
No	62	30 (48.4%)	Ref	
Yes	412	161 (39.1%)	0.68 (0.42–1.13)	0.136
Maternal education				
None	45	21 (46.7%)	Ref	0.267
Primary education	260	105 (40.4%)	0.77 (0.39–1.56)	
Secondary education	160	64 (40.0%)	0.76 (0.37–1.57)	
Postsecondary education	9	1 (11.1%)	0.14 (0.02–1.22)	
Maternal employment^c^				
Not formally employed	307	133 (43.3%)	Ref	
Employed	167	58 (34.7%)	0.70 (0.46–1.05)	0.081
Strata				
Blantyre urban	157	67 (42.7%)	Ref	<0.001
Lilongwe urban	101	38 (37.6%)	0.81 (0.74–0.89)	
North and central rural	117	51 (43.6%)	1.04 (0.75–1.44)	
South rural	99	35 (35.4%)	0.73 (0.56–0.96)	
Travel time				
<1 h	217	89 (41.0%)	Ref	0.469
1–2 h	175	71 (40.6%)	0.98 (0.74–1.31)	
>2 h	82	31 (37.8%)	0.87 (0.68–1.12)	

*Note*: Risk analysis of stunted growth restricted to CHEU contributing to multi‐variable logistic regression on stunted growth at age 24 months. CHEU who were accompanied by a guardian were excluded from the risk factor analysis.

Abbreviations: ART, antiretroviral therapy; ARV, antiretroviral; CI, confidence interval; CTX, co‐trimoxazole prophylaxis; HCC, HIV Care Clinic; OR, odds ratio; VL, viral load.

^a^ Among 474 CHEU with data available on diagnosis of an infectious disease, 18.4%, 28.3%, 4.6% and 0.63% were reported to have malaria, diarrhoea, pneumonia and/or meningitis diagnosis since the previous study visit, respectively.

^b^ Ever received antiretrovirals for a pregnancy before the most recent pregnancy with study child.

^c^ Not formally employed included mothers who self‐reported their employment as ‘housewife’.

**Table 3:** Factors associated with stunted growth at age 24 months by multi‐variable logistic regression

			Unweighted model		Weighted model^a^	
			Adjusted OR (95% CI)	*p* Value	Adjusted OR (95% CI)	*p* Value
	Total (*N*)	*n* (%) stunted	*N* = 474		*N* = 464	
Child age (months)	474	–	1.15 (0.99–1.35)	0.073	1.16 (0.97–1.38)	0.103
Child sex						
Male	232	104 (44.8%)	Ref		Ref	
Female	242	87 (36.0%)	0.70 (0.46–1.07)	0.054	0.70 (0.44–1.11)	0.128
Child low birthweight						
No	415	163 (38.8%)	Ref		*Ref*	
Yes	59	30 (50.9%)	1.61 (1.06–2.44)	0.025	*1.51 (0.81–2.83)*	*0.120*
Child optimal feeding in the past 7 days						
No	215	96 (44.7%)	Ref		Ref	
Yes	259	95 (36.7%)	0.70 (0.46–1.07)	0.098	0.70 (0.49–0.98)	0.039
Maternal employment^b^						
Not formally employed	307	133 (43.3%)	Ref		Ref	
Employed	167	58 (34.7%)	0.68 (0.44–1.05)	0.081	0.64 (0.44–0.95)	0.028
Child diagnosis of an infectious disease since previous study visit						
No	293	107 (36.5%)	Ref		*Ref*	
Yes	181	84 (46.4%)	3.35 (1.82–6.17)	<0.001	*1.32 (0.85–2.04)*	*0.142*
Received CTX at age 24 months						
No	96	50 (52.1%)	Ref		Ref	
Yes	378	141 (37.3%)	0.71 (0.41–1.24)	0.230	0.56 (0.41–0.77)	<0.001
Child diagnosis of an infectious disease * received CTX at age 24 months interaction			0.39 (0.16–0.90)	0.028	–	–
**Nonreceipt of CTX**						
No ID diagnosis	65	28 (43.1%)	Ref		Ref	
ID diagnosis	31	22 (71.0%)	3.35 (1.82, 6.17)	<0.001	–	–
**Receipt of CTX**						
No ID diagnosis	228	79 (34.7%)	Ref		Ref	
ID diagnosis	150	62 (41.3%)	1.29 (0.88–1.90)	0.192	–	–
Maternal suboptimal ART adherence in the past 30 days						
Adherent	444	173 (39.0%)	*Ref*		Ref	
Non‐adherent (missed ≥ 2 days)	30	18 (60.0%)	*2.16 (0.86–5.42)*	0.100	2.82 (1.10–7.25)	0.031
Maternal age						
<21 years	34	20 (58.8%)	*Ref*		Ref	
21‐34 years	332	128 (38.6%)	*0.50 (0.23–1.09)*	0.083	0.32 (0.15–0.65)	0.002
≥35 years	108	43 (39.8%)	*0.58 (0.23–1.47)*	0.247	0.40 (0.16–1.01)	0.051
Child ever hospitalised						
No	396	152 (38.4%)	*Ref*		Ref	
Yes	78	39 (50.0%)	*1.37 (0.88–2.13)*	0.162	1.64 (0.98–2.74)	0.061

*Note*: Unweighted and weighted multi‐variable risk analysis restricted to CHEU with complete data across exposure variables of interest; odds ratio adjusted for child age and sex. CHEU who were accompanied by a guardian were excluded from the multi‐variable regression.

Grey boxes with *italic* numbers denote the estimated odds ratio (95% CI) and Wald test *p*‐value for each covariate when added to the unweighted or weighted model successively. Discrepancy in sample sizes (*n* = 10) for the unweighted (*n* = 474) and weighted models (*n* = 464) was due to missing data in enrolment predictors used to estimate the predicted probabilities of follow‐up at 24 months.

Abbreviations: CI, confidence interval; CTX: co‐trimoxazole prophylaxis; ID. infectious disease; OR, odds ratio.

^a^ Weights were computed by predicting the probability of follow‐up at 24 months using logistic regression and enrolment characteristics (maternal characteristics: age, parity, timing of HIV diagnosis, timing of ART initiation, ART adherence, HIV viral load; infant characteristics: sex, age, exclusive breastfeeding, stunting, low birthweight, mother‐reported infant health, infectious disease diagnosis, enrolment in an HIV Care Clinic; and contextual factors: maternal employment and geographical location).

^b^ Not formally employed included mothers who self‐reported their employment as ‘housewife’.

In our multi‐variable analysis, being female was associated with a 30% (adjusted odds ratio [aOR]: 0.70 [0.46–1.07]) reduced odds of stunting than being male. Adjusting for child age and sex, CHEU optimal feeding in the past 7 days (aOR: 0.70 [0.46–1.07]) and maternal employment (aOR: 0.68 [0.44–1.05]) were weakly associated with reduced odds of stunting at age 24 months. Low birthweight was moderately associated with a 61% increased odds of stunted growth (aOR: 1.61 [1.06–2.44]) at 24 months. Child diagnosis of an infectious disease since the previous study visit was strongly associated with increased odds of stunting; however, this relationship was significantly modified by receipt of (*p* = 0.028). Among CHEU receiving CTX, diagnosis of an infectious disease was associated with a 29% increased odds of stunting than CHEU with no diagnosis (aOR: 1.29 [0.88–1.90]), whereas odds of stunting were 3.35 times increased in CHEU not receiving CTX (aOR: 3.35 [1.82–6.17]).

In our IPW multi‐variable analysis, which accounted for mother–child pairs who did not attend follow‐up at 24 months, results were similar (Table 3). In contrast to the unweighted analysis, maternal age, suboptimal ART adherence, and child ever hospitalised were retained and infant low birthweight and child infectious disease diagnosis were removed as their significance was above the pre‐defined threshold (*p* > 0.1). In the weighted model, child ever hospitalised was retained as a marker of morbidity but there was no evidence of an interaction between hospitalisations and CTX (*p* = 0.118), as observed in the unweighted model where the effect of CTX differed in those with and without infections. Where there were discrepancies between the covariates retained in the unweighted and weighted models, excluded variables were added to the models one at a time and coefficient estimates were consistent.

## 4 DISCUSSION

Using data from a prospective cohort study conducted in the context of high antenatal ART coverage and breastfeeding, we found that, even when HIV acquisition was successfully prevented, around 42% of CHEU were growth deficient at 24 months of age. We also identified various factors associated with stunting at 24 months, most notably low birthweight, infectious disease diagnosis, receipt of CTX, and optimal feeding.

Stunting typically begins in early infancy and continues throughout the first 2 years of life (Hoddinott et al., 2013; Victora et al., 2010). Our data highlight that linear growth faltering occurred early, with rates increasing between age 12 and 24 months, similar to findings from other Eastern and Southern African studies (Aizire et al., 2020; Evans et al., 2021; Fowler et al., 2021; Jumare et al., 2019; S. M. Le Roux et al., 2019; Ndiaye et al., 2021; Sudfeld et al., 2016). These rates are congruous with that reported in 2015–2016 in the general Malawian population, where stunting peaked at 42%–45% between the ages of 18 and 47 months (The DHS Programme, 2017). Compared to some sub‐Saharan African studies of CHEU, our prevalence estimates of underweight and stunting were higher (Aizire et al., 2020; S. M. Le Roux et al., 2019; Ndiaye et al., 2021; Sudfeld et al., 2016), but lower than others (Evans et al., 2021; Jumare et al., 2019), including the Zimbabwean SHINE trial where more than half of CHEU were stunted by age 18 months (Evans et al., 2021). Some studies have also reported a greater risk of stunting in CHEU than in children HIV‐unexposed (Aizire et al., 2020; Evans et al., 2021; S. M. Le Roux et al., 2019), although the relative contribution of HIV‐specific exposures remains unclear (Evans et al., 2021; S. M. Le Roux et al., 2019) and was not assessed in our analysis.

HIV‐specific exposures which may influence child growth and development include exposure to HIV proteins and glycoproteins, maternal immune compromise, and antiretroviral drugs *in utero* and via breastfeeding. In our study, 88% of CHEU were exposed to ART antenatally and 91% through breast milk. Although previous studies have observed an association between HIV/ART exposure and stunting (Ejigu et al., 2020; Evans et al., 2021; D. M. Le Roux et al., 2019), timing of exposure to maternal ART and maternal HIV viral load were not associated with stunting at age 24 months in our analysis, which found the strongest predictors of stunting to be universal risk factors, such as child optimal feeding.

In family settings, food insecurity often co‐exists with HIV (Wedderburn et al., 2019), and we found that 20% of mothers overall had MUAC measurements suggestive of being underweight, compared to 7% of women (aged 15–49 years) in Malawi (The DHS Programme, 2017). In a Malawian study, mothers with stunted children had lower levels of human oligosaccharides in breast milk at age 6 months, possibly due to lower quality breast milk resulting from maternal undernutrition (Charbonneau et al., 2016; D. M. Le Roux et al., 2019). Data on CHEU feeding practices and subsequent growth are limited, although inadequate complementary feeding increased the risk of low WAZ and LAZ among CHEU aged 12 and 18 months in Côte d'Ivoire (Becquet et al., 2006). In the SHINE trial, improved complementary feeding practices increased CHEU mean LAZ scores and reduced stunting prevalence (Prendergast et al., 2019), demonstrating that nutritional interventions have the potential in reversing early linear growth faltering. Our data showed higher rates of optimal feeding (55% in the past week) than overall in Malawi (where only 8% of children aged 6–23 months receive appropriate complementary feeding (The DHS Programme, 2017), but only 7% of CHEU were breastfed at age 24 months. In our risk analysis, non‐breast milk and exclusive breast milk feeding at study enrolment were associated with 30%–50% reduced odds of stunting at age 24 months. Optimal feeding at 24 months correlated with 30% reduced odds of stunting in our multi‐variable analysis, which together highlight the importance of adequate nutrition during infancy and early childhood for later growth. Despite this, we found stunting rates increased between 12 and 24 months, which may have been partially driven by poor weaning practices and subsequent nutritional deficiencies (Black et al., 2008). This underscores an ongoing need for prolonged breastfeeding promotion, nutrition education, and early nutritional intervention.

Low birthweight was reported in 12% of CHEU in our risk factor analyses, similar to population‐level estimates (The DHS Programme, 2017; United Nations Children's Fund, & WHO, 2019), and was moderately associated with a 63% increased odds of stunting at 24 months. These findings are consistent with it being a well‐established predictor of stunting (Black et al., 2013), and associated with lower WAZ and LAZ scores among CHEU elsewhere (Moseholm et al., 2019; Ramokolo et al., 2013). Several sub‐Saharan African studies have also demonstrated that CHEU have an increased risk of low birthweight (Bulterys et al., 1994; Ladner et al., 1998) and other adverse birth outcomes (e.g., preterm birth; Bulterys et al., 1994; Friis et al., 2007; Ndirangu et al., 2012) compared to children HIV‐unexposed, with some suggesting this risk persists in the context of lifelong ART and is higher with exposure to specific antiretroviral drugs (Fowler et al., 2016; Kourtis & Fowler, 2011; Malaba et al., 2017; Stringer et al., 2018; Zash et al., 2017). In Botswana, a mediation analysis of stunting attributed a 67% excess risk of stunting to low birthweight among CHEU aged 2+ years, (Sudfeld et al., 2016) highlighting that CHEU's greater risk of adverse birth outcomes compounds later risk for poor growth. We also found child diagnosis of infectious disease since the previous study visit was associated with an increased stunting risk, congruous with previous findings (Black et al., 2008; Dewey & Begum, 2011). Expanding evidence from high‐income and low‐ and middle‐income countries suggests CHEU have a higher risk of infectious diseases (Adler et al., 2015; Evans et al., 2016; Koyanagi et al., 2011; Slogrove et al., 2016) and hospitalisation compared to children HIV‐unexposed (Labuda et al., 2019; Powis et al., 2019; Slogrove et al., 2017), especially early life viral and bacterial respiratory infections (Cohen et al., 2016; D. M. Le Roux et al., 2015, 2019).

In our study, among CHEU with growth measured at 24 months, approximately 80% received CTX at that time point. Our data present strong evidence that CTX may be protective against stunting, contradictory to other findings (Daniels et al., 2019; Lockman et al., 2017; Pavlinac et al., 2021). In malaria‐endemic regions, CTX reduces the incidence of malaria among CHEU (Sandison et al., 2011), although its benefits in non‐malaria regions remain unclear. In a recent South African randomised control trial, a non‐malaria region, growth faltering did not differ by CTX at 12 months (Daniels et al., 2019). However, given its high malaria burden (The DHS Programme, 2017), the effect of CTX on growth may differ in Malawi. In our study, we observed no difference in child diagnosis of an infectious disease or ever being hospitalised by CTX, although CTX may have treated other unknown or incompletely treated infections in the first year of life (Pavlinac et al., 2021), potentially reducing the severity of infections and subsequent risk of stunting.

In Malawi, guidelines recommend that CHEU are registered with HCCs following delivery and attend monthly visits, where anthropometry, clinical monitoring, and HIV testing are provided alongside CTX prescription. Our data showed that HCC registration combined with receipt of CTX was associated with reduced odds of stunting by approximately 45% at age 24 months (*p* = 0.002). Additionally, our multi‐variable analysis revealed significant effect modification by receipt of CTX on the association between infectious disease diagnosis and stunting. This suggests that regular clinical care provided by the HCCs in combination with the provision of CTX may play an important role in optimising child growth, supporting current guidelines for CHEU follow‐up care in Malawi.

Our study has several limitations: since enrolment took place at under‐5 clinics, we may have missed women with serious pregnancy complications, acutely ill neonates, or higher‐risk infants not attending routine visits. Most measures were self‐reported by mothers with varying recall periods, which may carry a risk of bias. A substantial proportion of CHEU did not complete follow‐up to 24 months and differences in enrolment characteristics between those with incomplete and complete follow‐up were noted. Non‐attendance at 24 months was addressed using IPW in the multi‐variable model; estimates from weighted models were consistent and reassuring. However, weights were computed using enrolment characteristics which may not be the best predictors of follow‐up at 24 months and we cannot rule out the possibility the follow‐up data were missing not at random. Children who did not complete 24‐month follow‐up were less likely to attend HCCs and receive CTX, which was associated with reduced odds of stunting. As such, the prevalence estimate for stunting at 24 months should be interpreted as a minimum estimate for the subgroup of CHEU engaged in HIV care and who completed follow‐up. Although prevalence of stunting among CHEU at age 24 months was similar to national rates in Malawi, these estimates likely include children HIV‐exposed and HIV‐unexposed and may not be a true comparison population. Growth z‐scores were generated using a reference population of children from different ethnic and cultural backgrounds (World Health Organization, 2006), which may not be the most accurate representation of child growth in Malawi. Additionally, data on child age were only available in whole months which may have influenced prevalence estimates, although sensitivity analyses suggested the margin of error was negligible. As linear growth faltering is characterised by a progressive decline during the first 2 years of life, multi‐variable models at age 24 months were sufficient and captured cumulative stunting. Finally, without a control group of children HIV‐unexposed, we were unable to explore the effect of HIV exposure on stunting. Although our analysis identified the strongest predictors of stunting to be universal risk factors, this study was conducted in the context of high national malnutrition rates and may not have detected small differences by HIV‐specific exposures.

## 5 CONCLUSIONS

This is one of the first studies examining long‐term CHEU growth outcomes since the implementation of Option B+. Our findings demonstrate that over a third of CHEU were growth deficient at 24 months and identified various factors associated with stunting, which likely impact growth via distinct mechanisms. Interventions to improve linear growth among CHEU should address the multi‐faceted nature of their health environment, including prevention of infections and improved nutrition, alongside maternal ART prescription and follow‐up of mother–child pairs, to reduce any lasting impacts of early growth failure on long‐term health.

## AUTHOR CONTRIBUTIONS

Study design and methods were developed by Gabriela Toledo, Claire Thorne, Heather Bailey, and Siobhan Crichton. Gabriela Toledo conducted the analysis and wrote the manuscript. Claire Thorne, Heather Bailey, Siobhan Crichton, and Megan Landes supervised the work of Gabriela Toledo and Siobhan Crichton provided statistical support. Claire Thorne, Heather Bailey, Siobhan Crichton, Megan Landes, Monique van Lettow, Wezi Msungama, and Beth A. Tippett Barr reviewed and approved the manuscript for submission.

## CONFLICT OF INTEREST

The authors declare no conflict of interest.

## Supporting information

Supporting information.

Supporting information.

## Data Availability

The data that support the findings of this study are available from the Malawi Ministry of Health. Restrictions apply to the availability of these data, which were used under license for this study. Data are available from Beth Tippett Barr with the permission of the Malawi Ministry of Health.
