# Supplementary material for: Risk factors for stunting in children who are HIV‐exposed and uninfected after Option B+ implementation in Malawi
Source: Matern Child Nutr. 2022 Nov 9;19(1):e13451. doi: 10.1111/mcn.13451 (PMC9749602; doi:10.1111/mcn.13451)
Supplement: Supplementary file 1 — Supporting information. [file MCN-19-e13451-s001.docx]

| **Table S1.** Summary of child anthropometry by age | | | | | | |
| --- | --- | --- | --- | --- | --- | --- |
|  |  | **1-6 months** |  | **12 months** |  | **24 months** |
|  | n | Median [IQR] or N (%) | n | Median [IQR] or N (%) | n | Median [IQR] or N (%) |
| **Weight** † |  |  |  |  |  |  |
| Weight-for-age (WAZ) | 1,189 | -0.66 [-1.53, 0.17] | 661 | -0.42 [-1.13, 0.33] | 517 | -0.81 [-1.43, -0.11] |
| Underweight (WAZ< -2) |  | 192 (16.1%) |  | 53 (8.0%) |  | 50 (9.7%) |
| **Length** † |  |  |  |  |  |  |
| Length-for-age z-score (LAZ) | 1,119 | -2.15 [-3.32, -1.02] | 643 | -1.21 [-2.02, -0.31] | 517 | -1.68 [-2.66, -0.98] |
| Stunted (LAZ< -2) |  | 564 (50.4%) |  | 162 (25.2%) |  | 209 (40.4%) |
| **Weight** ‡ |  |  |  |  |  |  |
| Weight-for-age (WAZ) | 398 | -0.71 [-1.69, 0.07] | 398 | -0.42 [-1.13, 0.33] | 398 | -0.70 [-1.35, -0.11] |
| Underweight (WAZ< -2) |  | 69 (17.34) |  | 28 (7.04) |  | 32 (8.04) |
| **Length** ‡ |  |  |  |  |  |  |
| Length-for-age z-score (LAZ) | 370 | -2.00 [-3.26, -0.97] | 370 | -1.22 [-2.02, -0.38] | 370 | -1.68 [-2.65, -0.85] |
| Stunted (LAZ< -2) |  | 182 (49.19) |  | 93 (25.14) |  | 147 (39.73) |

† Child anthropometry estimated in CHEU with weight-for-age or length-for-age z-scores available at each study visit, respectively.

‡ Child anthropometry estimated in CHEU with complete weight-for-age and/or length-for-age z-scores available across study visits (i.e. complete-cases).
