# Supplementary material for: Risk factors for stunting in children who are HIV‐exposed and uninfected after Option B+ implementation in Malawi
Source: Matern Child Nutr. 2022 Nov 9;19(1):e13451. doi: 10.1111/mcn.13451 (PMC9749602; doi:10.1111/mcn.13451)
Supplement: Supplementary file 2 — Supporting information. [file MCN-19-e13451-s002.docx]

**Table S2.** Sensitivity analysis of child anthropometry by age

|  |  | **1-6 months** |  | **12 months** |  | **24 months** |
| --- | --- | --- | --- | --- | --- | --- |
|  | N | Median [IQR] or N (%) | N | Median [IQR] or N (%) | N | Median [IQR] or N (%) |
| **Weight** |  |  |  |  |  |  |
| Weight-for-age (WAZ) | 1,189 | -0.12 [-0.93, 0.76] | 661 | -0.32 [-1.03, 0.44] | 517 | -0.72 [-1.35, -0.04] |
| Underweight (WAZ< -2) |  | 103 (8.66) |  | 41 (6.20) |  | 45 (8.70) |
| **Length** |  |  |  |  |  |  |
| Length-for-age z-score (LAZ) | 1,119 | -1.38 [-2.78, -0.37] | 643 | -0.97 [-1.8, -0.06] | 517 | -1.78 [-2.61, -0.93] |
| Stunted (LAZ< -2) |  | 420 (37.53) |  | 153 (23.79) |  | 200 (38.68) |

Sensitivity analysis was conducted as child age was only available in whole months. Growth z-scores were recalculated using child age minus two weeks to estimate minimum prevalence rates for underweight and stunting by child age.
